# Exosome‐Based Cartilage‐Targeted Delivery System: Strategies and Applications

**DOI:** 10.1002/smsc.202500436

**Published:** 2025-11-21

**Authors:** Yao Wang, Bo Chen, Haodong Zhu, Zhenyu Sun

**Affiliations:** ^1^ Department of Orthopedic Surgery Shanghai Ninth People's Hospital Shanghai Jiao Tong University School of Medicine Shanghai 200011 China; ^2^ Department of Orthopedic Surgery The First Affliated Hospital Zhejiang University School of Medicine Hangzhou 310003 China

**Keywords:** cartilage‐targeted delivery, exosome, intervertebral disc degeneration, osteoarthritis, rheumatoid arthritis

## Abstract

With the ageing of the global population, cartilage‐related diseases, such as osteoarthritis (OA) and intervertebral disc degeneration (IVDD), have increasingly become significant social problems threatening human health. Therefore, targeted therapy for cartilage is becoming more and more promising. Exosomes, natural cellular derivatives, have emerged as promising therapeutic vectors owing to their inherent biocompatibility, superior biomatrix penetration capabilities, and therapeutic efficacy in cartilage regeneration. Precise targeting of cartilage tissues can be achieved through specific construction strategies, showing potential for treating cartilage‐related diseases. However, a review of cartilage‐targeted exosomes is still lacking. Previous studies have merely categorized chondrocytes under the broader group of osteocytes, regarding them only as a supplementary component of bone‐targeted therapy, or have been limited to a single modification technique. This review specifically focus on cartilage‐targeted exosomes, systematically integrating two modification methods—direct surface modification and parental cell engineering—and highlights translational applications in disease contexts. This article elaborates in detail on the construction strategies of cartilage‐targeted exosomes and explores their application progress in related diseases such as OA and IVDD, aiming to provide a reference for further research and clinical translation in this field.

## Graphical abstract

Construction strategies and applications of cartilage‐targeting exosomes.

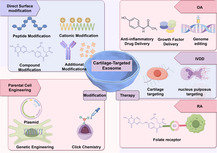

## 1 Introduction

Cartilage, as a vital component of the skeletal system, plays a critical role in maintaining the normal function of bones and joints.^[^ ^1^ ^]^ However, due to its limited self‐repair capacity, cartilage tissue often fails to regenerate after injury, leading to the development of degenerative diseases such as osteoarthritis (OA) and intervertebral disc degeneration (IVDD).^[^ ^2^, ^3^ ^]^ Conventional therapeutic approaches—including pharmacological treatments, physical therapies, and surgical interventions—are often ineffective in directly targeting cartilage tissue.^[^ ^2^, ^4^ ^]^ Therefore, the development of cartilage‐specific drug delivery systems is of paramount importance.

Cartilage tissue consists of chondrocytes embedded within an extracellular matrix (ECM) secreted by these cells. The cartilage ECM is composed of collagen, proteoglycans, and inorganic components.^[^ ^5^, ^6^ ^]^ Proteoglycans, such as chondroitin sulfate, form glycosaminoglycans (GAGs) by binding to hyaluronic acid.^[^ ^7^ ^]^ These densely packed GAG chains are rich in sulfated carbohydrates and carry a net negative charge under physiological conditions.^[^ ^7^, ^8^ ^]^ Negatively charged proteoglycans undergo electrostatic interactions with the positively charged regions of collagen, forming stable complexes.^[^ ^6^, ^8^ ^]^ The dense structure of the chondrocyte ECM serves as a key biological barrier for drug delivery,^[^ ^5^ ^]^ which hinders drug absorption for conventional therapeutic approaches, such as intra‐articular injection. Therefore, the development of a drug delivery method capable of overcoming the cartilage biological barrier aligns with clinical needs to a certain extent. Targeted delivery of drugs or gene‐editing molecules to cartilage tissue—by leveraging the electrostatic interactions, chemical binding, and ligand‐receptor binding principles inherent to cartilage tissue—represents an approach with significant clinical value.

Exosomes, as naturally occurring nanovesicles, possess excellent biocompatibility and stability and can carry various bioactive molecules such as proteins, nucleic acids, and lipids.^[^ ^9^ ^]^ In recent years, they have garnered significant attention in the field of drug delivery. Specifically, cartilage‐targeted exosomes can undergo functional modifications through specific engineering strategies, enabling them to specifically target cartilage tissue, achieve precise drug delivery and prolonged retention, and provide novel therapeutic approaches for cartilage‐related diseases.^[^ ^10^ ^]^ First, as endogenous biological nanocarriers derived from cells, exosomes inherently exhibit lower immunogenicity and higher biocompatibility compared with synthetic liposomes (which may trigger complement activation) and polymeric nanoparticles (some of which carry cytotoxic risks due to their degradation products).^[^ ^10^ ^]^ Second, the unique nanoscale size (≈30–150 nm) and surface protein characteristics (e.g., integrins, tetraspanins) of exosomes enable them to penetrate the dense, avascular cartilage ECM more effectively—outperforming larger liposomal formulations or polymeric nanoparticles, which tend to get trapped in the collagen/proteoglycan network of the matrix.^[^ ^11^ ^]^ Third, exosomes’ ability to interact with surface receptors on chondrocytes and synovial cells facilitates their prolonged retention in the cartilage microenvironment. In contrast, synthetic nanocarriers, due to their lack of specific cell‐binding motifs, are usually cleared more rapidly.^[^ ^12^ ^]^ Interestingly, the exosome membrane contains various proteins, lipids, and carbohydrates that serve as modification targets.^[^ ^12^, ^13^, ^14^ ^]^ For example, exosomes express membrane proteins such as CD9, CD63, CD81, and Lamp2b.^[^ ^10^, ^12^, ^13^, ^14^, ^15^ ^]^ By fusing the extracellular domains of these proteins with genes encoding cell‐targeted peptides, these peptides can be displayed on the exosome surface.^[^ ^9^, ^15^ ^]^ Lipid molecules can be modified through exchange or binding with lipid analogs to alter surface properties, while carbohydrates can adjust surface charge and bioactivity via glycosylation.^[^ ^12^ ^]^ Finally, parental cells producing exosomes can undergo pretreatment or genetic engineering to indirectly modify the derived exosomes.^[^ ^16^, ^17^ ^]^

**Figure 1:**
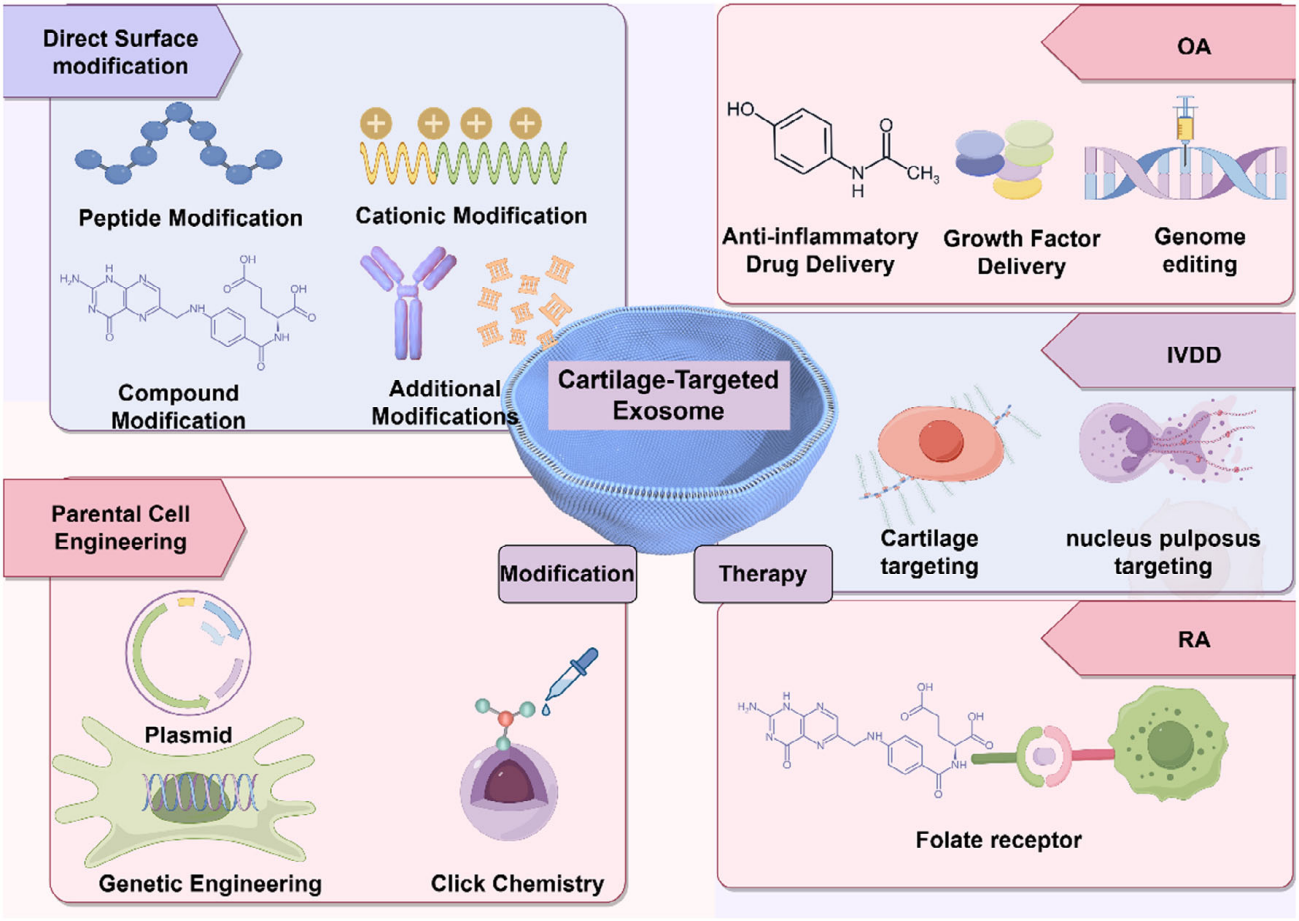
Exosome‐based cartilage‐targeted delivery system.

In summary, exosomes demonstrate superior modifiability and compatibility compared to traditional cartilage‐targeted biomaterials. Although exosomes have attracted considerable research attention in recent years, comprehensive reviews focusing on cartilage‐targeted exosomes remain lacking. Previously, relevant reviews have habitually classified chondrocytes as a subset of osteocytes, merely treating cartilage‐targeting exosomes as a supplementary component of bone‐targeting exosomes.^[^ ^18^ ^]^ This review specifically focuses on cartilage‐targeting exosomes and explores in detail their construction strategies and applications in disease treatment. Furthermore, this review systematically integrates chemical modification and parental cell engineering strategies from the perspectives of both exosomes themselves and the parental cells that produce exosomes. This avoids the limitation of previous studies, which only one‐sidedly discussed the direct modification of exosomes while overlooking indirect modification approaches. Finally, we delve into the disease‐specific translational challenges in cartilage‐related disorders, particularly those encountered in OA, IVDD, and rheumatoid arthritis (RA) (**Figure** **1**). The analysis aims to provide researchers with new insights for further developing bone‐targeted exosomes and their applications in cartilage‐related pathologies.

## 2 Constructive Strategies of Cartilage‐Targeted Exosomes

The development of cartilage‐targeted exosomes primarily employs two distinct engineering approaches: (1) direct surface functionalization and (2) parental cell reprograming. The former strategy involves postisolation modifications through chemical conjugation or physical adsorption to engineer exosomal membranes. Conversely, the latter approach modifies plasma membranes of exosome‐producing cells, leveraging the intrinsic inheritance of membrane constituents and surface molecules during exosome biogenesis.^[^ ^18^ ^]^

### 2.1 Direct Surface Modification

Direct surface modification represents one of the most widely employed functionalization strategies for exosomes. Through chemical reactions or physical interactions, specific targeting ligands—including polypeptides, nucleic acids, and small molecular compounds—can be effectively modified on the exosomal surface, thereby significantly enhancing the exosomes’ specific binding capability to cartilage tissue.^[^ ^12^, ^14^, ^15^ ^]^ These targeting ligands typically interact with specific receptors or molecules within cartilage tissue, enabling the achievement of cartilage‐targeted delivery of exosomes.

#### 2.1.1 Peptide Modification

**Figure 2:**
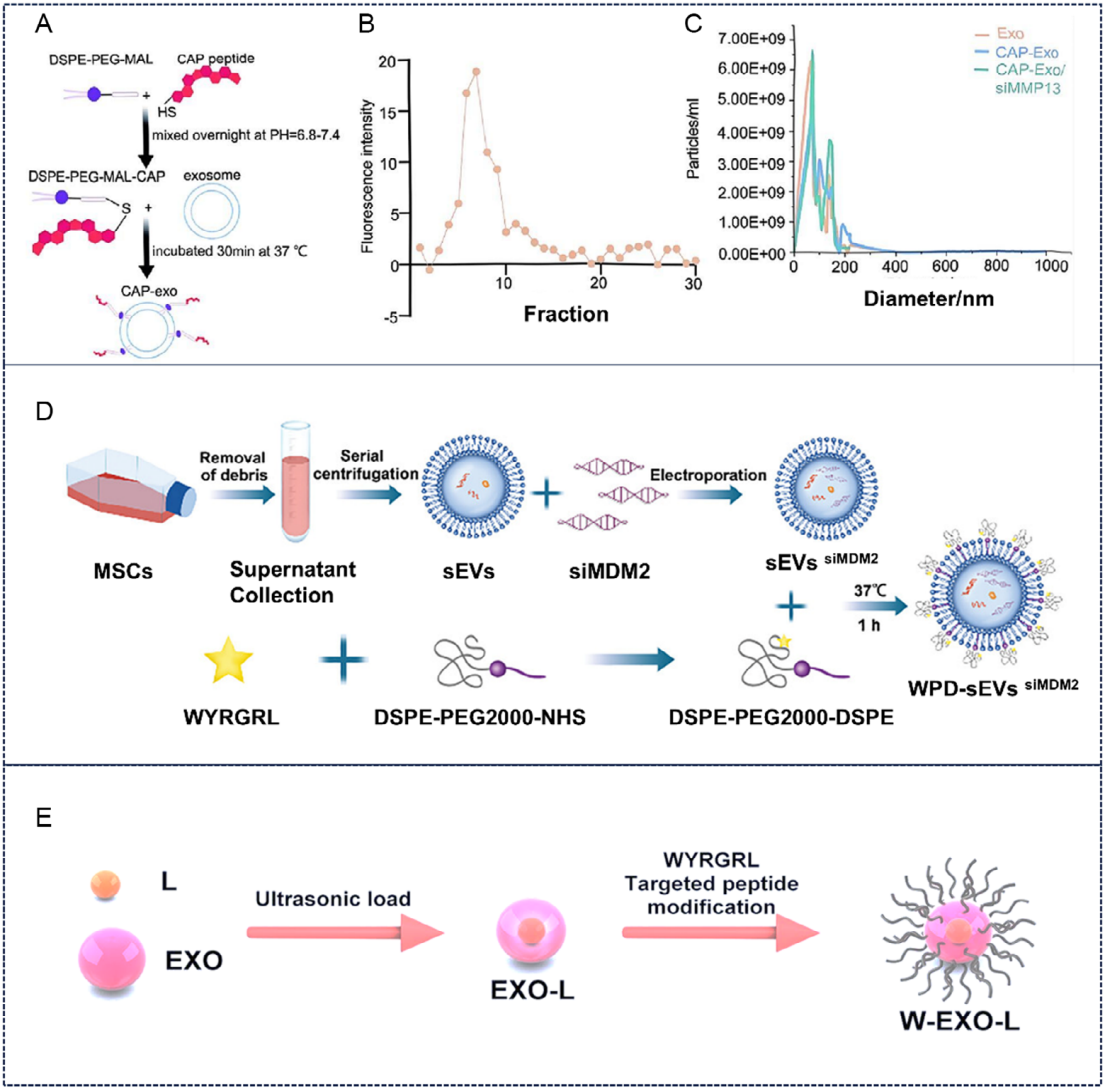
Peptide modification of cartilage‐targeted exosomes. A–C) Schematic illustration of CAP peptides modification of cartilage‐targeted exosomes. Reproduced with permission.^[^ ^21^ ^]^ Copyright 2024, Elsevier. D) Schematic illustration of WYRGRL peptides modification of exosomes loaded with siMDM2 and their flow cytometry and Zeta potential analysis. Reproduced with permission.^[^ ^24^ ^]^ Copyright 2025, John Wiley and Sons. E) Construction of WYRGRL peptide‐modified exosomes. Reproduced with permission.^[^ ^23^ ^]^ Copyright 2023, Biomed Central.

The chondrocyte‐affinity peptide (CAP), a widely employed targeting peptide, was identified via phage display technology by Pi et al. as the sequence DWRVIIPPRPSA.^[^ ^19^ ^]^ This peptide exhibits high specificity for chondrocytes across species. CAP can be anchored to exosome membranes through covalent conjugation using chemical crosslinkers such as EDC/NHS to bridge carboxyl/amino groups on the peptide with amino or sulfhydryl groups on exosomal membrane proteins.^[^ ^19^ ^]^ Alternatively, hydrophobic CAP peptides may be inserted into the lipid bilayer of exosomes via hydrophobic interactions. Yan et al. employed three strategies—click chemistry (using dibenzocyclooctyne, DBCO), membrane insertion (via DSPE‐PEG), and enzymatic ligation (sortase A)—to engineer CAP‐modified exosomes (CAP‐exo).^[^ ^20^ ^]^ While all methods preserved exosome size, enzymatic modification yielded the highest cellular uptake efficiency. Zhang et al. utilized lipid insertion to modify exosome surfaces with CAP, creating chondrocyte‐targeted exosomes (CAP‐Exo), which were then loaded with MMP13‐targeted siRNA to form CAP‐Exo/siMMP13 (**Figure** **2**A–C). In an ACLT‐induced OA rat model, intra‐articular injection of CAP‐Exo/siMMP13 effectively reduced MMP13 levels while increasing collagen COL2A1 and aggrecan content in cartilage.^[^ ^21^ ^]^

Through phage display screening using the FUSE5 system, Rothenfluh et al. identified a hexameric linear random peptide (WYRGRL) demonstrating specific binding affinity to type II collagen, the principal component of cartilaginous matrix.^[^ ^22^ ^]^ This engineered peptide exhibited potent cartilage tissue penetration capabilities and sustained retention properties within articular tissues. Building upon this discovery, Wan and colleagues developed an innovative drug delivery system through methacrylic anhydride‐mediated conjugation of WYRGRL peptides to exosomal surface amino groups, thereby enhancing chondrocyte‐targeted specificity.^[^ ^23^ ^]^ Subsequently, these drug‐loaded exosomes were encapsulated within methacrylated gelatin (GelMA) microspheres, achieving significantly prolonged intra‐articular retention duration and reduced clearance rate in OA models.^[^ ^23^ ^]^ Feng et al. successfully encapsulated siRNA (siMDM2) into mesenchymal stem cell (MSC)‐derived exosomes and subsequently modified these exosomes with a cartilage‐targeted peptide, WYRGRL‐PEG2K‐DSPE (Figure 2D–F).^[^ ^24^ ^]^ The experimental results demonstrated that this multifunctional modification significantly enhanced the cellular uptake of MSC‐derived small extracellular vesicles (MSC‐sEVs) by chondrocytes, thereby improving their antiaging efficacy. Notably, the WYRGRL peptide modification was found to augment the cartilage‐penetrating capacity of the exosomes while prolonging their retention time within articular joints.

Beyond the established CAP and WYRGRL peptides, emerging evidence suggests additional cartilage‐targeted peptide candidates. Cheung et al. identified two 12‐mer peptides (RLDPTSYLRTFW and HDSQLEALIKFM) through third‐round phage library biopanning against murine chondrocytes.^[^ ^25^ ^]^ Quantitative analysis using enzyme‐linked immunosorbent assay (ELISA) revealed comparable cartilage ECM binding capacities between these sequences, with particular affinity for proteoglycan components. In parallel screening efforts, Mi et al. isolated HAP‐1, a synovium‐targeted transduction peptide facilitating cellular internalization in synovial cell lines.^[^ ^26^ ^]^ Notably, current research remains scarce regarding the application potential of these peptides in exosome‐based delivery systems.

Interestingly, according to the statistical results in our tables (**Table** **1** and **2**), studies using CAP peptides are far more numerous than those using WYRGRL peptides. Compared with CAP, WYRGRL contains hydrophobic amino acids such as tryptophan (Trp) and tyrosine (Tyr), which pose greater challenges in synthesis and are more costly.^[^ ^19^, ^22^, ^23^ ^]^ In addition, WYRGRL peptides rely on type II collagen for anchoring.^[^ ^23^ ^]^ The presence of collagenases in OA joints may also affect its targeting efficacy. This may explain why the utilization rate of CAP is higher than that of WYRGRL.

**Table 1:** Construction strategies of cartilage‐targeted exosomes.

Modification methods	Ligand types	Therapeutic cargo	Limitations	Reference
Peptide modification	CAP	siRNA	Small molecular weight	[19, 20, 21]
	WYRGRL	siRNA	High synthesis cost	[22, 23, 24]
	RLDPTSYLRTFW/HDSQLEALIKFM	/	/	[25]
	HAP‐1	/	/	[26]
Cationic modification	Avidin	IL‐1RA	Depend on biotin	[27, 28, 29]
	Oligolysine	CCR2 miRNA	The stability is relatively poor.	[30]
	Succinylated chitosan (Sch)	Kartogenin TGF‐β1	Depend on chemical modification reactions	[31]
Compound modification	Folic acid (FA)	Dexamethasone sodium phosphate	Excessive supplementation of folic acid poses health risks.	[41, 42]
	Chondroitin sulfate (ChS)	Aceclofenac	Insufficient targeting ability	[43]
Other modification	Nucleic acid aptamer	Dasatinib	Depend on SELEX screening	[45, 46]
	Antibody	Nicotinamide	High production cost	[47]
Genetic engineering	CAP	sgRNA	sgRNA has poor safety	[49, 50]
	Cavin‐2	miRNAs proteins	/	[51, 52]
Click chemistry	DBCO‐DS	miRNA	The procedure is complex	[53, 54, 55]

**Table 2:** Applications of cartilage‐targeted exosomes in diseases.

Disease models	Delivery method	Functions	Clinical readiness	Reference
OA	ChS‐Lipo	Inhibited inflammation by delivering aceclofenac	Preclinical study	[43]
	CAP‐Exo	Scavenged ROS by delivering SOD3	Preclinical study	[61]
	Sch ‐Exo	Promoted cartilage repair by delivering TGF‐β1	Preclinical study	[31]
	Col2A1‐Exo	Promoted cartilage repair by delivering TGF‐β1	Preclinical study	[47]
	CAP‐Exo	Reversed chondrocyte senescence by delivering siRNA (si‐STING)	Preclinical study	[71]
	CAP‐Exo	Inhibited the expression of MMP13 by delivering antisense oligonucleotides	Preclinical study	[20]
	CAP‐Exo	Promoted cartilage regeneration by delivering miR‐199a‐3p	Preclinical study	[60]
IVDD	CAP‐Exo	Alleviated chondrocyte oxidation by delivering Nrf2	Preclinical study	[79]
	CAP‐Exo	Alleviated intracellular iron accumulation and reduced the level of ROS by delivering tanshinone IIA	Preclinical study	[80]
	Cavin2‐Exo	Alleviated pyroptosis of NPs by delivering antioxidant proteins	Preclinical study	[46]
	Cavin2‐Exo	Alleviated pyroptosis in NP cells through the delivery of antioxidant proteins	Preclinical study	[52]
	Cavin2‐Exo	Delayed IVDD by regulating the dkk2‐mediated mitochondrial unfolded protein response	Preclinical study	[51]
	Cavin2‐Exo	Reduced DNA damage in NPs by delivering plasmids encoding pluripotency‐related genes	Preclinical study	[82]
	Exo derived from blood	The endogenously present anti‐inflammatory factors have alleviated IVDD.	Phase I clinical trial	[81]
RA	FA‐ Exo	Alleviated joint inflammation by delivering dexamethasone sodium phosphate	Preclinical study	[42]
	FA‐GDEVs	targeted M1 macrophages by engineering exosomes derived from ginger	Preclinical study	[87]
	Oligolysine‐MEX	Clear cfDNA by engineering exosomes derived from M2 macrophages	Preclinical study	[30]

#### 2.1.2 Cationic Surface Modification

**Figure 3:**
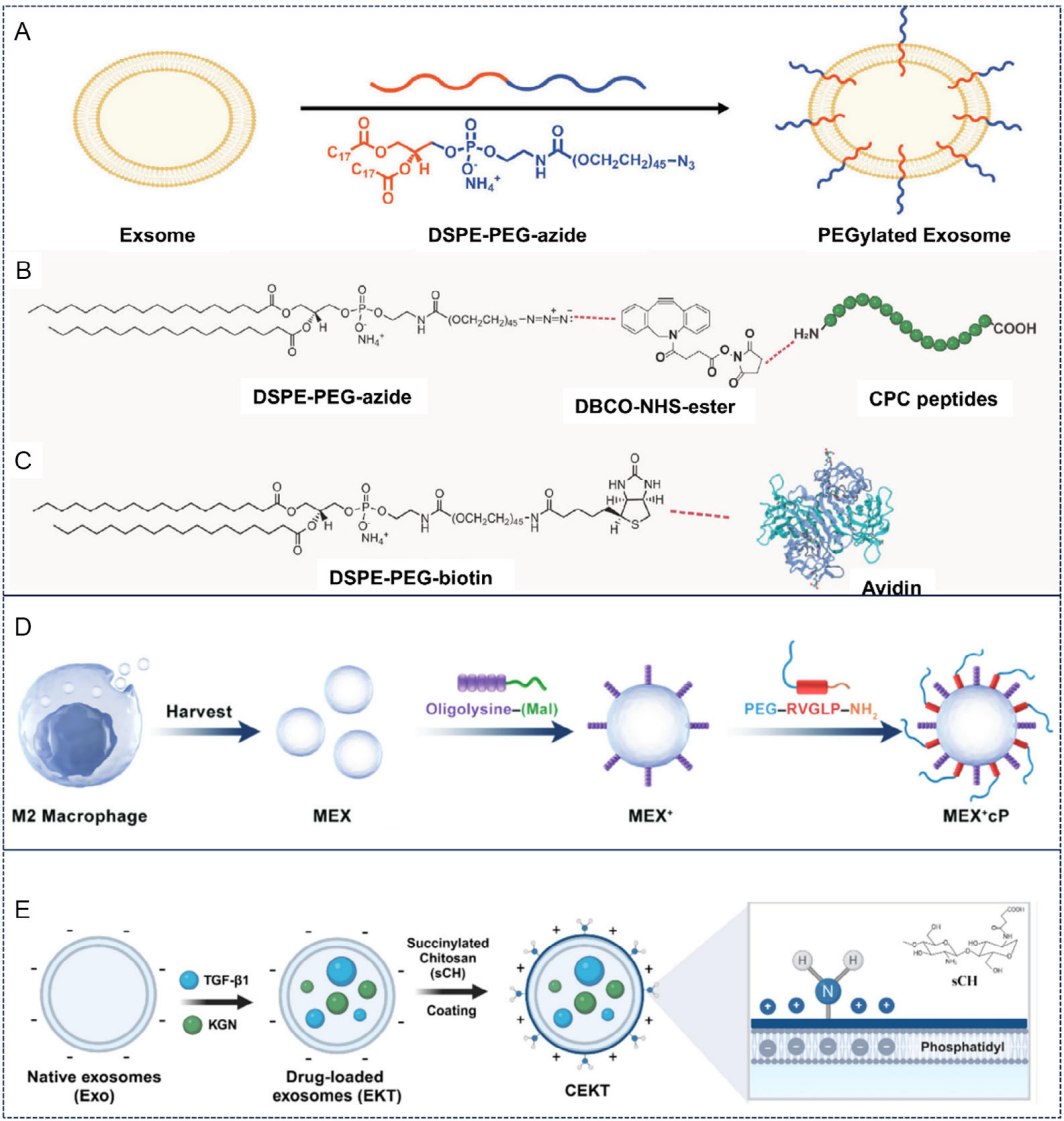
Cationic surface modification of cartilage‐targeted exosomes. A–C) Schematic illustration of Avidin modification of exosomes. Reproduced with permission.^[^ ^29^ ^]^ Copyright 2024, John Wiley and Sons. D) Schematic illustration of oligolysine modification of exosomes derived from M2 macrophage. Reproduced with permission.^[^ ^30^ ^]^ Copyright 2023, John Wiley and Sons. E) Construction of sCH modified exosomes. Reproduced with permission.^[^ ^31^ ^]^ Copyright 2025, Elsevier.

As established in introductory sections, the electronegative microenvironment of cartilage tissue under physiological conditions originates from its glycosaminoglycan (GAG) composition. This biophysical property enables targeted cartilage delivery through electrostatic interactions mediated by surface functionalization of exosomes with cationic moieties. Avidin, a 60 kDa tetrameric glycoprotein comprising four subunits with high biotin‐binding affinity, exhibits strong cationic characteristics at physiological pH (isoelectric point (pI) 10–10.5).^[^ ^27^ ^]^ Pioneering work by Bajpayee et al. demonstrated avidin's cartilage‐penetrating capacity: this 7 nm‐diameter cationic glycoprotein effectively permeates intact cartilage matrix through electrostatic binding with anionic GAGs.^[^ ^28^ ^]^ Building upon this mechanism, Zhang et al. engineered cationic exosomes by anchoring avidin to exosomal surfaces via biotin‐avidin conjugation (**Figure** **3**A–C).^[^ ^29^ ^]^ These modified vesicles demonstrated full‐thickness penetration in early‐stage arthritic cartilage and efficient mRNA delivery to chondrocytes in deep tissue layers.

Alternative cationic modifications for cartilage‐targeted exosomes include polycationic amino acids and chitosan derivatives. Wang et al. enhanced articular targeting of M2 macrophage‐derived exosomes through oligolysine modification, achieving concurrent clearance of cell‐free DNA (cfDNA) and therapeutic efficacy in RA models (Figure 3D).^[^ ^30^ ^]^ In a separate approach, Tu et al. developed succinylated chitosan (sCH)‐modified exosomes encapsulating Kartogenin (KGN) and transforming growth factor‐β1 (TGF‐β1) (Figure 3E). The sCH coating conferred sufficient surface charge to overcome cartilage electronegativity barriers, enabling deep matrix penetration and site‐specific drug release.^[^ ^31^ ^]^

Notably, avidin itself is a neutral protein, which requires the indirect introduction of cationic groups via the “biotin‐avidin system”.^[^ ^32^ ^]^ In vivo experiments have shown that while this system enables stable specific binding, the multistep centrifugation/purification involved in the preparation process reduces the recovery rate of exosomes.^[^ ^32^ ^]^ In contrast, cationic amino acids are essential amino acids for the human body. After modifying exosomes, their degradation products are natural amino acids—substances that are nonimmunogenic and noncytotoxic.^[^ ^33^ ^]^ However, they exhibit poor stability and are prone to slow hydrolysis in the physiological environment (e.g., synovial fluid with a pH of ≈7.3–7.4 and containing proteases).^[^ ^34^ ^]^ Currently, research on cationic amino acid modification remains in the exploratory stage. Chitosan is derived from shrimp and crab shells (a natural and renewable resource).^[^ ^35^ ^]^ Its derivatives have a mature synthesis process and extremely low application costs. Furthermore, a large number of chitosan‐based excipients and sprays are currently used in clinical practice.^[^ ^36^, ^37^ ^]^ This indicates that chitosan‐modified exosomes hold great potential for clinical application in the future.

#### 2.1.3 Compound Modification

**Figure 4:**
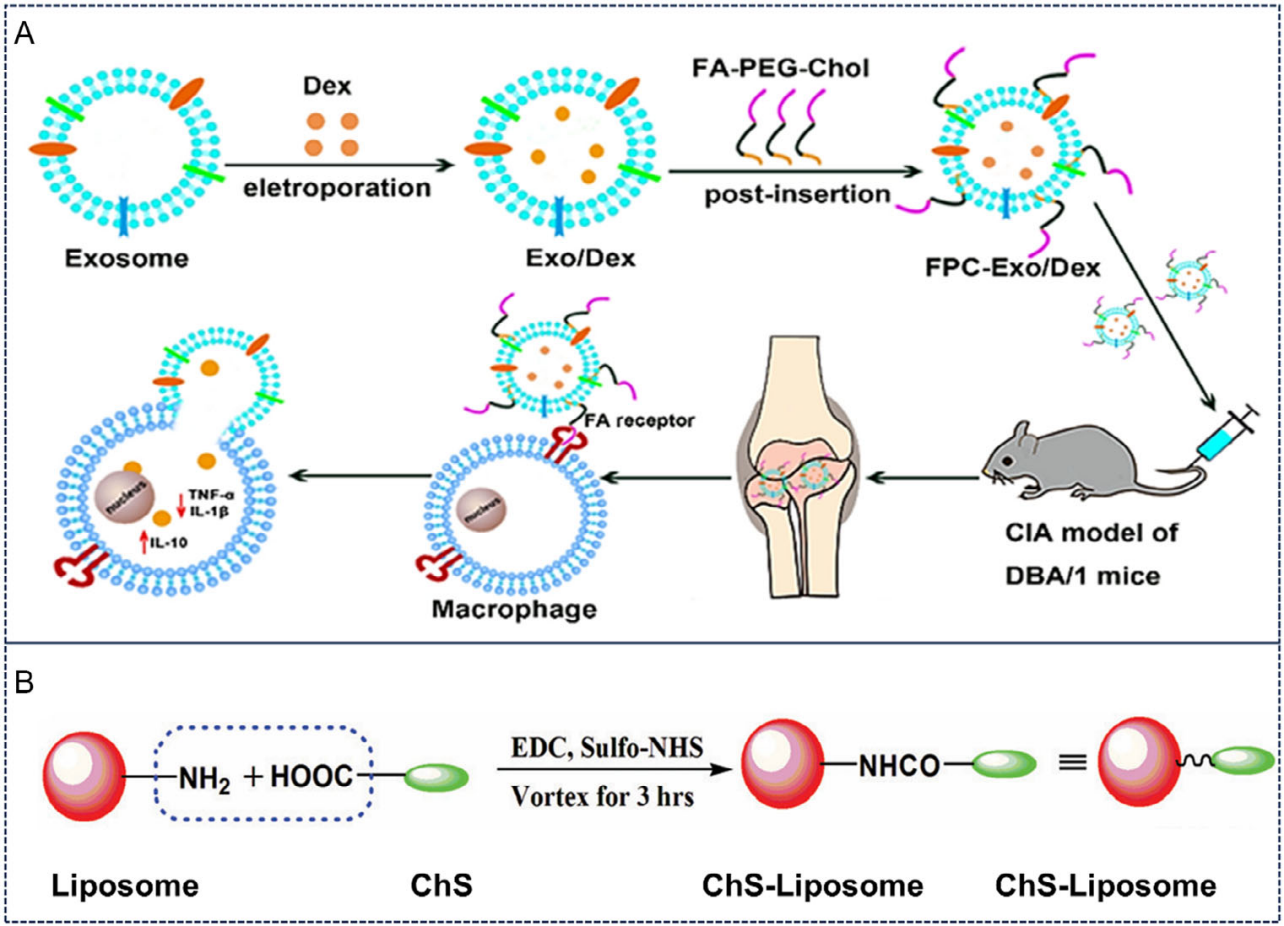
Compound modification of cartilage‐targeted exosomes. A) Schematic illustration of FA modification of exosomes. Reproduced with permission.^[^ ^42^ ^]^ Copyright 2020, John Wiley and Sons. B) Schematic illustration of ChS modification of Liposome. Reproduced with permission.^[^ ^43^ ^]^ Copyright 2014, Taylor Francis.

Certain bioactive compounds, including folic acid (FA), chondroitin sulfate (ChS), and hyaluronic acid (HA), demonstrate inherent articular or chondral affinity through selective binding to tissue‐specific receptors.^[^ ^38^, ^39^, ^40^ ^]^ Recent advances in exosome engineering have capitalized on these ligands for surface functionalization to enhance therapeutic targeting. Notably, folate receptors (FRs) exhibit marked overexpression on activated macrophages within inflammatory microenvironments.^[^ ^41^ ^]^ This pathophysiological feature has been exploited by conjugating folic acid to exosomal membranes, thereby enabling precise targeting of cartilaginous inflammation. In one illustrative application, Yan et al. engineered FR‐targeted exosomes through surface modification with a folate‐polyethylene glycol (PEG)‐cholesterol ternary complex (**Figure** **4**A). Subsequent anti‐inflammatory drug loading via electroporation yielded functionalized exosomes that demonstrated site‐specific accumulation in arthritic murine joints, resulting in significantly attenuated synovial inflammation.^[^ ^42^ ^]^

Contemporary research has further elucidated ChS's targeting mechanisms, revealing its ability to interact with type II collagen, CD36 receptors, and other chondrocyte‐associated membrane proteins.^[^ ^38^ ^]^ Building upon this targeting mechanism, Bishnoi and colleagues developed ChS‐conjugated liposomes for intra‐articular delivery of aceclofenac in OA treatment (Figure 4B). Postintravenous administration, quantitative biodistribution analysis revealed substantially greater accumulation of ChS‐modified liposomes in knee joints compared to their unmodified counterparts.^[^ ^43^ ^]^ This targeting paradigm has been extrapolated to exosome engineering, where ChS surface functionalization leverages native cartilage binding affinity to enhance both targeting specificity and tissue retention duration of therapeutic exosomes. Correspondingly, hyaluronic acid binds to CD44 receptors on macrophage surfaces, enabling targeted accumulation at inflammatory sites.^[^ ^40^ ^]^

#### 2.1.4 Additional Modifications

**Figure 5:**
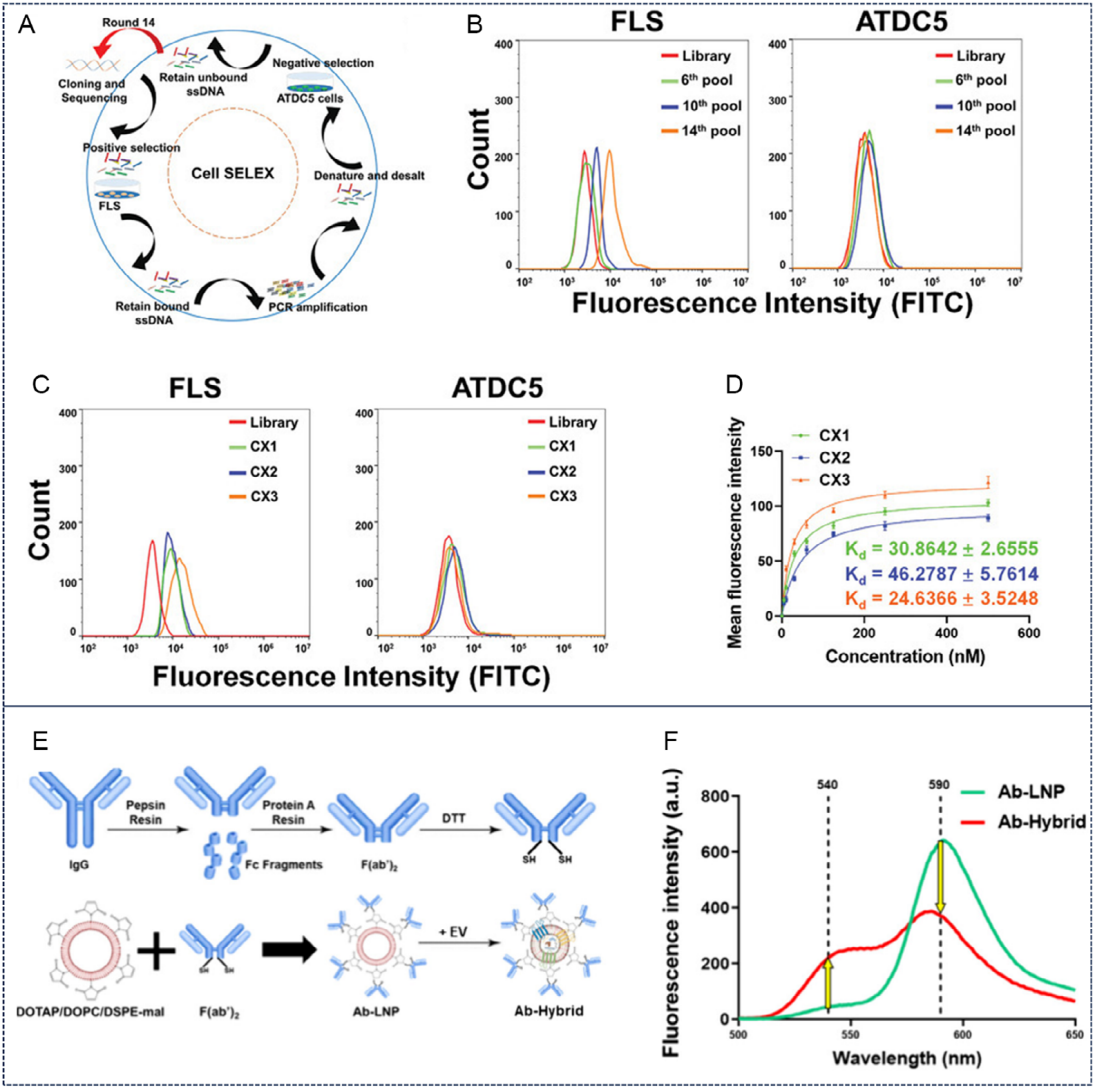
Additional modifications of cartilage‐targeted exosomes. A–D) The screening process of CX3 and its affinity detection for FLS. Reproduced with permission.^[^ ^46^ ^]^ Copyright 2022, John Wiley and Sons. E,F) Schematic illustration of Col2A1 antibody modification of exosomes. Reproduced with permission.^[^ ^47^ ^]^ Copyright 2024, American Chemical Society.

Nucleic acid aptamers and antibodies are frequently employed for exosome surface engineering. Aptamers are oligonucleotide fragments typically isolated from nucleic acid libraries through in vitro selection via the Systematic Evolution of Ligands by EXponential Enrichment (SELEX) technique.^[^ ^44^ ^]^ These low‐molecular‐weight biomolecules exhibit unique tertiary structures enabling specific binding to peptides, nanoparticles, or entire cells.^[^ ^45^ ^]^ Their stringent molecular recognition capabilities and binding affinities confer high target specificity. Chen et al. identified a synovium‐targeted aptamer (CX3) through SELEX screening (**Figure** **5**A–E). Building upon this discovery, they engineered CX3‐modified liposomes for targeted delivery of dasatinib to eliminate senescent synovial cells in OA. Compared with unmodified liposomes, CX3‐functionalized drug carriers demonstrated enhanced synovial accumulation, reduced cartilage degradation, and lower OARSI scores in murine models.^[^ ^46^ ^]^

Protein antibodies have also been utilized for cartilage‐targeted exosome modifications. For instance, Kim et al. developed stable hybrid vesicles through ethanol‐mediated fusion between tonsil‐derived MSC exosomes and collagen II (Col2A1) antibody‐conjugated liposomes (Figure 5F–G). These hybrids demonstrated superior cartilage‐targeted efficiency and prolonged retention in a destabilized medial meniscus‐induced rat OA model.^[^ ^47^ ^]^

### 2.2 Parental Cell Engineering for Exosomal Modification

Beyond direct surface functionalization, exosome membranes can be indirectly modified through bioengineering of their progenitor cells—a process termed parental cell reprograming. During exosome biogenesis, these nanovesicles inherently inherit membrane constituents and surface molecules from their parental cells. When progenitor cells undergo genetic engineering or chemical modifications (e.g., introduction of click chemistry moieties), such alterations are autonomously incorporated into exosomal membranes.^[^ ^18^ ^]^ A representative study by Wang et al. demonstrated that overexpression of FUT6 membrane protein in engineered cells resulted in its stable presentation on secreted exosomes. Through ligand‐receptor interaction mechanisms, FUT6‐bearing exosomes achieved bone‐targeted efficacy by binding counter‐ligands expressed on bone marrow endothelial cells, with in vivo studies showing enhanced skeletal accumulation compared to naïve exosomes.^[^ ^48^ ^]^

#### 2.2.1 Genetic Engineering

**Figure 6:**
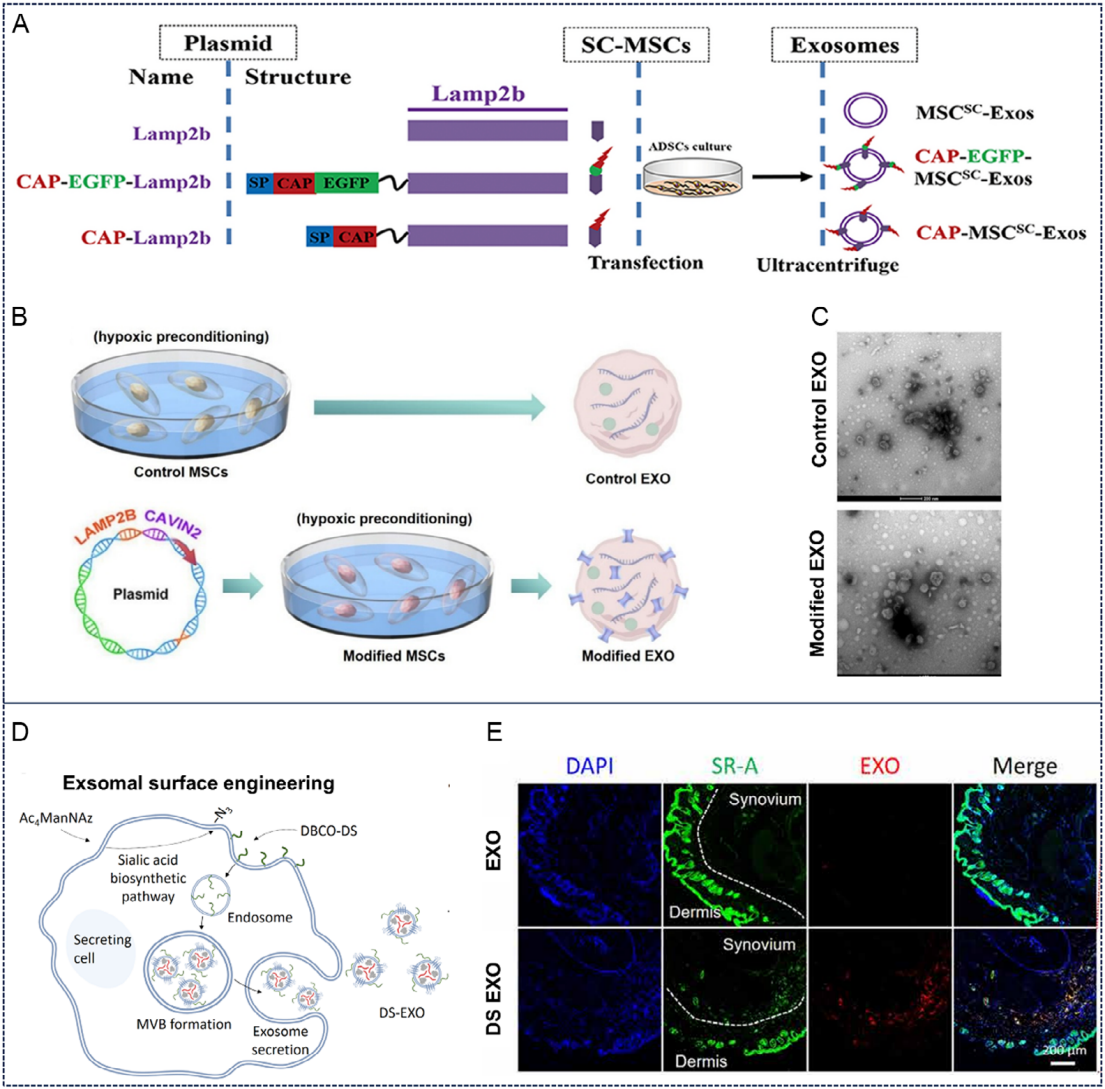
Exosomes modification based on genetic engineering and click chemistry. A) Transfection of CAP‐EGFP‐Lamp2b plasmid into MSCs and collection of their exosomes. Reproduced with permission.^[^ ^60^ ^]^ Copyright 2023, Biomed Central. B,C) MSCs were transfected with CAVIN2‐ Lamp2b plasmid and electron microscopy images of its extracellular vesicles. Reproduced with permission.^[^ ^51^ ^]^ Copyright 2021, American Chemical Society. D,E) Synthesis of DS‐decorated MSC‐EXO (DS‐EXO) and its distribution in inflammatory joints of the collagen‐induced arthritis mice. Reproduced with permission.^[^ ^55^ ^]^ Copyright 2021, American Association for the Advancement of Science.

This approach fundamentally modulates exosomal targeting capacity through genomic integration of cartilage‐homing proteins/peptides in parental cells, enabling endogenous incorporation of these motifs into secreted exosomes. As previously discussed, targeting peptides (e.g., CAP, WYRGRL) represent promising cartilage‐specific ligands.^[^ ^10^ ^]^ Genetic engineering allows covalent display of such peptides on exosomal surfaces by fusing their coding sequences with endogenous exosomal membrane protein genes (e.g., Lamp2b, tetraspanins CD9/CD63/CD81).^[^ ^13^, ^14^, ^16^ ^]^ Notably, exosomal membrane proteins—particularly lysosome‐associated membrane protein LAMP‐2B and tetraspanin family members—serve as ideal anchoring scaffolds (**Figure** **6**A).^[^ ^49^ ^]^ Liang et al. employed CRISPR/Cas9‐mediated genetic fusion of chondrocyte‐affinity peptide CAP with Lamp2b in parental cells, yielding exosomes with membrane‐embedded CAP motifs. Subsequent fusion of these modified exosomes with liposomes generated hybrid vesicles demonstrating enhanced chondrocyte‐targeted capacity in degenerative joint models.^[^ ^50^ ^]^

Emerging evidence suggests that specific genetic modifications beyond targeting peptide integration can confer cartilage‐targeted therapeutic potential to exosomes. Mechanistic studies reveal that Cavin‐2, a caveolae‐associated protein, mediates enhanced endocytic uptake of exosomes by nucleus pulposus cells.^[^ ^51^ ^]^ Genetic upregulation of Cavin‐2 in parental cells significantly amplifies the endocytic efficiency of engineered exosomes, thereby potentiating their therapeutic efficacy in IVDD (Figure 6B,C).^[^ ^51^ ^]^ Liao et al. implemented CRISPR/Cas9‐mediated genetic engineering in MSCs to generate Cavin‐2‐enriched exosomes. These modified vesicles demonstrated targeted delivery to degenerated nucleus pulposus cells, effectively attenuating cellular apoptosis and mitigating IDD progression in rodent models.^[^ ^52^ ^]^

#### 2.2.2 Click Chemistry

Current engineering strategies for exosomal modification through genetic engineering of parental cells or direct chemical functionalization present inherent limitations. Genetic modification approaches exhibit suboptimal efficiency in primary cell systems such as MSCs, whereas chemical conjugation methods require complex purification protocols that often compromise exosomal yield and impair vesicle functionality.^[^ ^53^, ^54^ ^]^ This technological gap has spurred interest in bioorthogonal metabolic engineering of parental cells. Representative work by Dong et al. demonstrated a metabolic glycoengineering strategy to develop surface‐modified exosomes with enhanced inflammatory joint targeting (Figure 6D,E).^[^ ^55^ ^]^ Specifically, adipose‐derived MSCs (ADSCs) were cultured with tetraacetylated N‐azidoacetyl‐d‐mannosamine (Ac4ManNAz) to biosynthetically incorporate azide groups onto membrane glycoproteins through the sialic acid metabolic pathway. Subsequent strain‐promoted azide‐alkyne cycloaddition enabled covalent conjugation of dibenzocyclooctyne‐modified dextran sulfate (DBCO‐DS) to azide‐functionalized ADSCs. The engineered exosomes leveraged dextran sulfate's specific binding affinity for scavenger receptor class A (SR‐A), a macrophage surface marker pathologically overexpressed in arthritic joints. These metabolically engineered exosomes demonstrated enhanced joint‐targeted capability while maintaining native vesicle integrity.^[^ ^55^ ^]^

However, compared with click chemistry, the biosynthetic system of genetic engineering is more amenable to standardization, and genetic engineering offers greater advantages in terms of long‐term stability.^[^ ^12^ ^]^ Furthermore, genetic engineering has a clearer regulatory pathway—it can draw on the regulatory experience of gene therapy products such as CAR‐T (Chimeric Antigen Receptor T‐cell Therapy) to accelerate the Investigational New Drug Application (IND)/New Drug Application (NDA) filing process.^[^ ^56^ ^]^ Furthermore, genetic engineering of parental cells hardly affects their exosome yield.^[^ ^13^ ^]^ Considering Good Manufacturing Practice (GMP) requirements and clinical translation needs, genetic engineering modification exhibits significant advantages. Similarly, in our tables (Table 1 and 2), the majority of studies are still conducted based on genetic engineering.

## 3 Applications in Cartilage‐Related Diseases

Cartilage‐targeted exosomes have emerged as innovative delivery vehicles for managing cartilage‐related pathologies, including OA, IVDD, and RA. These engineered nanovesicles demonstrate enhanced therapeutic precision through site‐specific delivery of pharmacologic agents to articular cartilage and synovial compartments, thereby amplifying therapeutic efficacy while minimizing systemic toxicity.

### 3.1 OA

OA manifests as a whole‐joint heterogenous disorder characterized by progressive cartilage degradation, synovitis, subchondral bone remodeling, and periarticular soft tissue alterations.^[^ ^57^ ^]^ Current pharmacologic interventions focus on multimodal symptom management through early‐stage, personalized therapeutic strategies.^[^ ^58^ ^]^ Cartilage‐targeted exosomes address OA's molecular complexity by delivering therapeutic cargo (anti‐inflammatory agents, growth factors, gene regulators) directly to chondrocytes.^[^ ^59^ ^]^

#### 3.1.1 Anti‐Inflammatory Drug Delivery

**Figure 7:**
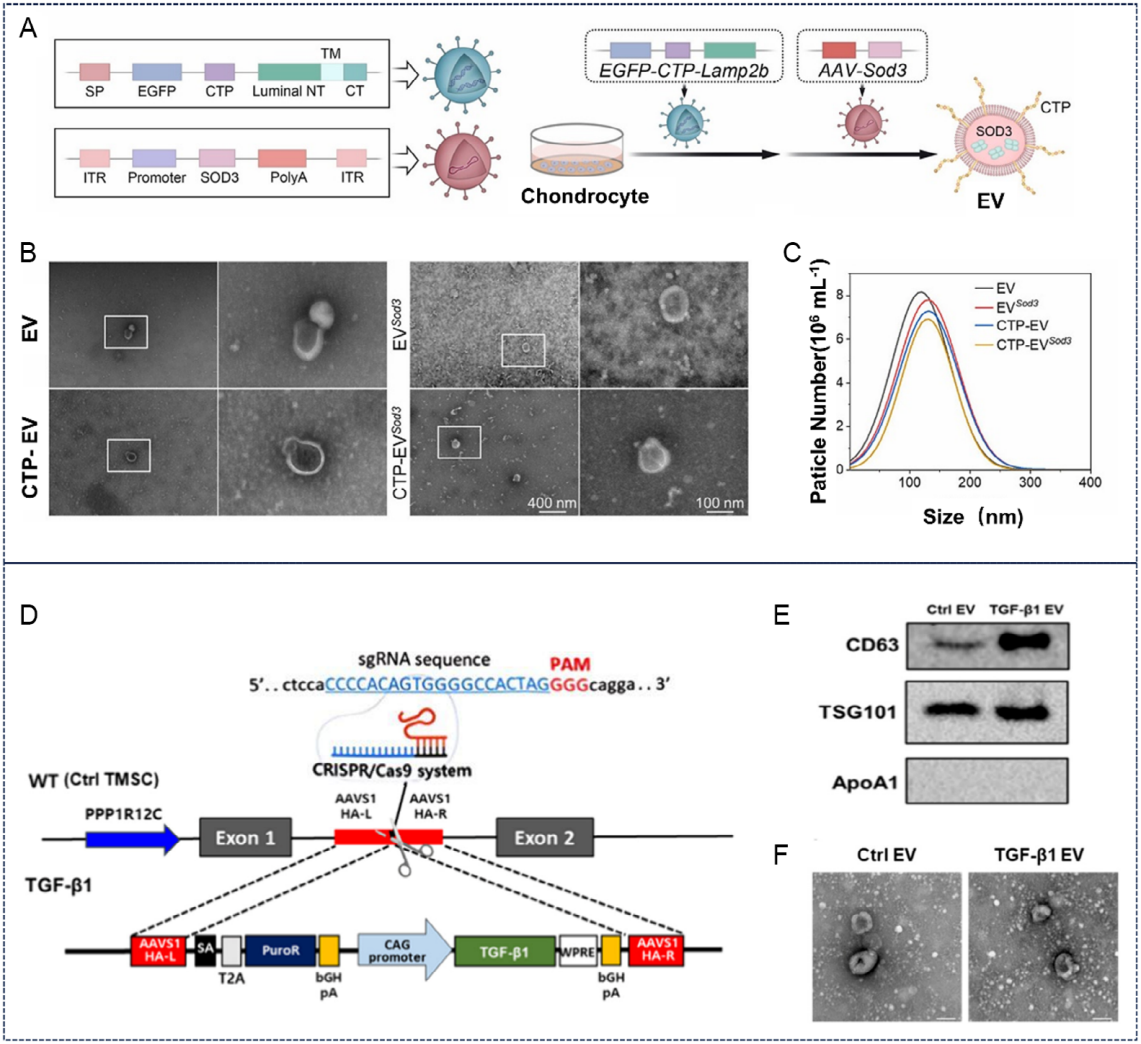
Cartilage‐targeted exosomes deliver anti‐inflammatory drugs and cytokines for OA therapy. A–C) CAP‐modified cartilage‐targeted exosomes loaded with SOD3 for osteoarthritis therapy. Reproduced with permission.^[^ ^62^ ^]^ Copyright 2025, Elsevier. D–F) Overexpression of TGF‐β1 in cartilage‐targeted exosomes for OA therapy. Reproduced with permission.^[^ ^47^ ^]^ Copyright 2021, American Association for the Advancement of Science.

The pathogenesis and progression of OA are closely associated with inflammatory responses within the joint cavity.^[^ ^58^ ^]^ Loading anti‐inflammatory drugs into cartilage‐targeted exosomes enables precise drug delivery and prolonged retention, thereby effectively alleviating joint inflammation and pain.^[^ ^60^ ^]^ Studies have demonstrated that anti‐inflammatory drugs delivered via cartilage‐targeted exosomes exhibit significant therapeutic effects in OA models. Bishnoi et al. utilized ChS‐modified liposomes loaded with aceclofenac, which demonstrated favorable therapeutic outcomes in knee OA following intravenous administration.^[^ ^43^ ^]^ Additionally, superoxide dismutase 3 (SOD3) enhances the scavenging capacity of chondrocytes against reactive oxygen species (ROS).^[^ ^61^ ^]^ Wu et al. transfected and selected chondrocytes stably expressing the CTP‐Lamp2b fusion peptide along with SOD3, subsequently isolating extracellular vesicles (CTP‐EV‐SOD3) via differential centrifugation (**Figure** **7**A–C). CTP‐EV‐SOD3 exhibited robust activation of the SOD3‐APOE signaling pathway within the joint, restoring chondrocyte antioxidant capacity and cholesterol homeostasis while mimicking the therapeutic effects of exercise, thus demonstrating promising translational potential.^[^ ^62^ ^]^

#### 3.1.2 Growth Factor Delivery

Growth factors such as insulin‐like growth factor‐1 (IGF‐1) and transforming growth factor‐β (TGF‐β) play pivotal roles in cartilage repair and regeneration.^[^ ^63^, ^64^, ^65^ ^]^ Loading these growth factors into cartilage‐targeted exosomes enables their precise delivery and efficient expression within cartilage tissue, thereby promoting chondrocyte proliferation and differentiation as well as ECM synthesis (Figure 6D,E).^[^ ^66^ ^]^ Researchers developed a cartilage‐targeted delivery system (Gel‐CEKT) by encapsulating TGF‐β1 and kartogenin into exosomes, followed by surface modification with succinylated chitosan to confer positive charge.^[^ ^31^ ^]^ The exosomes were subsequently crosslinked with oxidized chondroitin sulfate (oCS) and decellularized Wharton's jelly (WJ) to form a stable hydrogel. Experimental results demonstrated that Gel‐CEKT could maintain stable retention in the dynamic joint environment and effectively repaired the cartilage‐bone interface in a rat cartilage defect model, providing an integrated solution combining targeted delivery with sustained repair.^[^ ^31^ ^]^ Growth differentiation factor‐5 (GDF‐5), a crucial regulator of cartilage development, has also been explored for cartilage regeneration.^[^ ^67^ ^]^ Zheng et al. pretreated synovial mesenchymal stem cells (SMSCs) with GDF‐5 and isolated GDF‐5‐containing exosomes, which were then incorporated into a methacrylated hyaluronic acid (HAMA) scaffold to enhance endogenous cartilage regeneration.^[^ ^68^ ^]^ In another approach, Kim et al. employed CRISPR/Cas9 to overexpress TGF‐β1 in tonsil‐derived mesenchymal stem cells (TMSCs). The collected exosomes were hybridized with Col2A1 antibody‐modified liposomes to achieve cartilage‐specific TGFβ1 delivery.^[^ ^47^ ^]^ This hybrid system demonstrated superior cartilage repair and anti‐inflammatory effects in OA lesions.

#### 3.1.3 Gene Therapy

**Figure 8:**
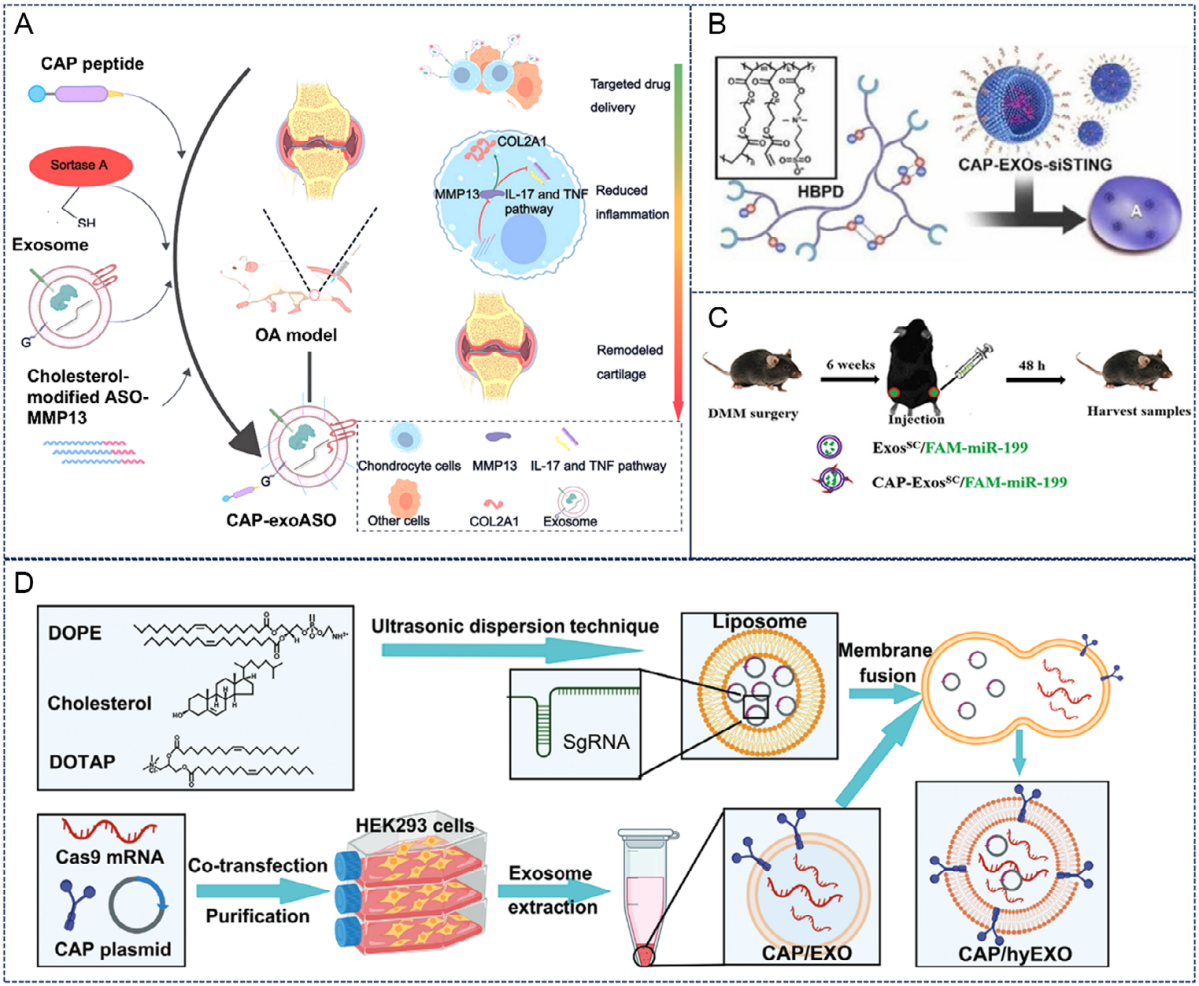
Gene‐modified tools delivered by cartilage‐targeted exosomes for OA therapy. A) CAP‐modified exosomes delivering ASO (MMP13‐targeted) for osteoarthritis therapy. Reproduced with permission.^[^ ^20^ ^]^ Copyright 2024, John Wiley and Sons. B) CAP‐modified exosomes delivering siSTING for OA therapy. Reproduced with permission.^[^ ^71^ ^]^ Copyright 2025, Elsevier. C) CAP‐modified exosomes delivering miR‐199 for OA therapy. Reproduced with permission.^[^ ^60^ ^]^ Copyright 2023, Biomed Central. D) Chondrocyte‐targeted exosomes deliver sgRNA (FGF18) to Osteoarthritic Mouse Chondrocytes for CRISPR‐Cas9 Gene Editing. Reproduced with permission.^[^ ^75^ ^]^ Copyright 2024, John Wiley and Sons.

Gene therapy represents a fundamental therapeutic strategy for OA by enabling precise genetic modifications through cartilage‐targeted exosomes loaded with gene‐editing tools such as plasmids and small interfering RNA (siRNA).^[^ ^69^, ^70^ ^]^ This approach facilitates targeted delivery of therapeutic agents to chondrocytes for specific gene regulation. Li et al. developed an injectable lubricating hydrogel encapsulating exosomes (EXOs) containing STING‐inhibitory siRNA (si‐STING), which incorporated cartilage‐targeted peptide (CAP) to achieve both articular lubrication and senescence microenvironment remodeling (**Figure** **8**B). In vitro and in vivo studies demonstrated that this hydrogel/exosome delivery system significantly reduced joint wear, reversed chondrocyte senescence, and suppressed OA progression, presenting a novel therapeutic approach.^[^ ^71^ ^]^ Antisense oligonucleotides (ASOs), composed of short single‐stranded nucleic acids, represent an emerging oligonucleotide therapy that specifically binds target RNA through sequence complementarity. Recognizing matrix metalloproteinase 13 (MMP13) as the primary enzyme responsible for cartilage degradation, Yan et al. engineered CAP‐modified exosomes to deliver MMP13‐targeted ASOs, effectively suppressing MMP13 expression in chondrocytes (Figure 8A).^[^ ^20^, ^72^ ^]^ MicroRNA delivery has also demonstrated therapeutic potential.^[^ ^73^ ^]^ Zhao et al. achieved cartilage‐specific miR‐199a‐3p delivery by fusing CAP with the exosomal membrane protein Lamp2b (Figure 8C). Intra‐articular administration of CAP‐Exos loaded with miR‐199a‐3p significantly enhanced cartilage regeneration in OA mice.^[^ ^60^ ^]^ Fibroblast growth factor 18 (FGF18), a crucial yet frequently silenced gene in OA, plays vital roles in chondrocyte proliferation and differentiation during cartilage regeneration.^[^ ^74^ ^]^ To achieve precise in vivo editing of FGF18, Chen et al. transfected and screened HEK293 cells stably expressing CAP/EGFP‐Lamp2b and Cas9 plasmids, subsequently isolating CAP/EXO exosomes from these cells via ultracentrifugation.^[^ ^75^ ^]^ Through membrane fusion techniques, they hybridized CAP/EXO with sgFGF18‐loaded liposomes to construct hybrid exosomes (CAP/FGF18‐hyEXO) carrying the FGF18 gene‐editing system (Figure 8D). Cellular experiments demonstrated that CAP/FGF18‐hyEXO significantly upregulated FGF18 expression in chondrocytes, confirming the effective gene regulatory capacity of CRISPR/Cas9‐based cartilage‐targeted exosomes for FGF18 modulation. This approach combines viral vector‐free gene delivery with tissue‐specific targeting, presenting promising therapeutic potential for cartilage regeneration.^[^ ^75^ ^]^

### 3.2 Intervertebral Disc Degeneration (IVDD)

The intervertebral disc (IVD) is composed of nucleus pulposus cells, fibroblastic cells, and chondrocytes. The nucleus pulposus constitutes the central region of the IVD, primarily consisting of water, ECM, and nucleus pulposus cells.^[^ ^76^ ^]^ The annulus fibrosus surrounds the nucleus pulposus as a fibrous tissue, mainly formed by fibroblasts and fibrochondrocytes.^[^ ^77^ ^]^ The cartilaginous endplate (CEP) is located at the superior and inferior margins of the IVD, functioning as a nutritional barrier that supplies oxygen and metabolic substrates to the nucleus pulposus via endplate vasculature.^[^ ^77^ ^]^ CEP degeneration is a critical factor in IVDD, characterized by irreversible chondrocyte death.^[^ ^76^ ^]^ The development of IVD‐targeted exosomes represents a promising therapeutic approach for IVDD.^[^ ^78^ ^]^ Current strategies for constructing targeted exosomes against IVDD primarily involve the use of homing peptides to target chondrocytes and Cavin‐2 to nucleus pulposus cells.

#### 3.2.1 Targeting Chondrocytes

**Figure 9:**
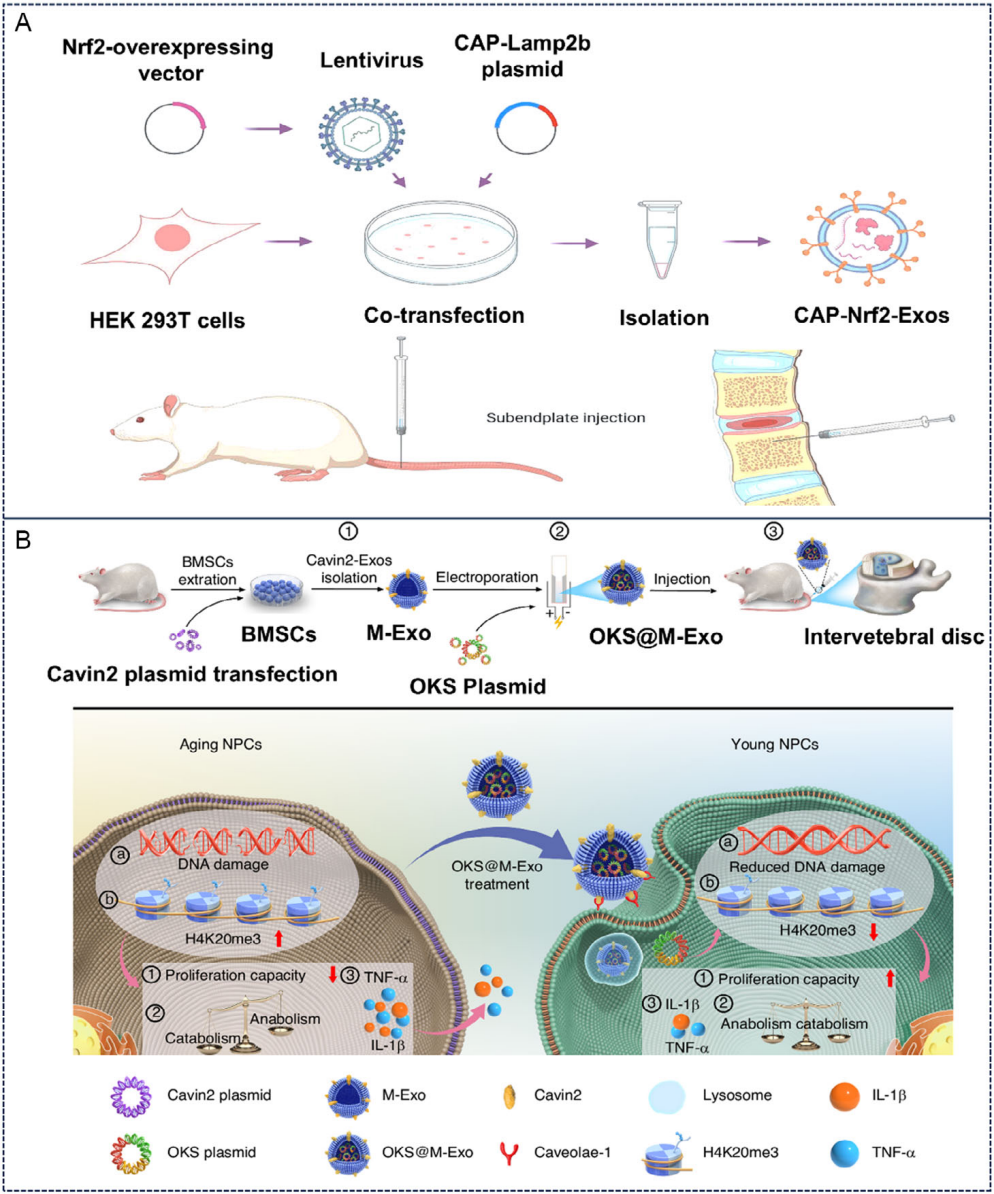
Cartilage‐targeted exosomes for IVDD therapy. A) CAP‐modified exosomes delivering Nrf2 for IVDD therapy. Reproduced with permission.^[^ ^79^ ^]^ Copyright 2024, John Wiley and Sons. B) CAVIN2‐modified exosomes delivering OKS plasmid for IVDD therapy. Reproduced with permission.^[^ ^82^ ^]^ Copyright 2025, Springer Nature.

Lin et al. established in vitro and in vivo models of CEP degeneration using lipopolysaccharide (LPS) and engineered gene‐modified exosomes (CAP‐Nrf2‐Exos) expressing CAP on their surface while carrying the antioxidant transcription factor nuclear factor erythroid 2‐related factor 2 (Nrf2).^[^ ^79^ ^]^ These chondrocyte‐targeted exosomes were generated by transfecting HEK293T cells with CAP‐Nrf2 overexpression plasmids (**Figure** **9**A). Following sub‐endplate injection, the therapeutic efficacy of these engineered exosomes was validated through radiological and histological analyses. Zhan et al. utilized CAP‐modified exosomes (CAP‐sEXOs) loaded with tanshinone IIA and incorporated them into a CaCO3/chitosan hydrogel. CAP‐sEXOs mitigated intracellular iron accumulation and reactive oxygen species (ROS) levels by suppressing the expression of hypoxia‐inducible factor‐2α (HIF‐2α) and transferrin receptor 1 (TfR1).^[^ ^80^ ^]^

#### 3.2.2 Targeting Nucleus Pulposus (NP)

Natural exosomes may also possess a certain degree of targeting ability. Researchers have initiated a Phase I clinical trial (NCT04849429) by directly injecting autologous platelet‐rich plasma (PRP) enriched with blood‐derived exosomes into the NP of patients’ intervertebral discs.^[^ ^81^ ^]^ This trial has verified the potential of natural blood‐derived exosomes for the treatment of IVDD. Liao et al. engineered parental stem cells to generate exosomes expressing Cavin‐2. The Cavin‐2‐modified exosomes effectively restored the uptake efficiency of NP cells for exosomes.^[^ ^52^ ^]^ These exosomes alleviated pyroptosis in NP cells through the delivery of antioxidant proteins, thereby mitigating cellular damage and degeneration. Lu et al. developed an engineered exosome‐hydrogel composite system by incorporating Cavin‐2‐modified exosomes into methacrylated silk fibroin hydrogel as a high‐efficiency delivery vehicle.^[^ ^51^ ^]^ This system significantly delayed IVDD by regulating the dkk2‐mediated mitochondrial unfolded protein response. Ma et al. targeted senescent NP cells in degenerative discs using Cavin‐2‐modified exosomes to deliver plasmids encoding pluripotency‐associated genes (OKS@M‐Exo), effectively reducing DNA damage and restoring the proliferative capacity and metabolic homeostasis of senescent NP cells (Figure 9B).^[^ ^82^ ^]^ Apart from Cavin‐2 modification, few alternative NP‐targeted strategies have been explored in the context of exosomes. For instance, Chen et al. employed SELEX technology to develop the nucleic acid aptamer RD4, which exhibits specific binding affinity for NP cells.^[^ ^83^ ^]^ RD4 was conjugated to PLGA polymer carriers for miRNA delivery to NP cells. In summary, current exosome‐based NP‐targeted approaches remain limited, highlighting the need to explore additional modification strategies.

### 3.3 RA

RA is a multisystem autoimmune disease characterized by chronic, polyarticular, and erosive synovitis.^[^ ^84^ ^]^ The affected joints exhibit marked synovial hyperemia, edema, and substantial effusion into the articular cavity, accompanied by pannus formation ‐ a vascular granulation tissue rich in capillaries that leads to articular cartilage destruction and subchondral bone resorption.^[^ ^85^ ^]^ Targeted therapeutic approaches for RA remain predominantly focused on anti‐inflammatory interventions.^[^ ^86^ ^]^

#### 3.3.1 Targeting Chondrocytes

Alleviating cartilage inflammation is of great value for the treatment of RA. Therefore, studies have been conducted to develop exosomes targeting RA for cartilage. Wang et al. reported a novel cartilage‐targeted delivery system based on M2‐type macrophage‐derived exosomes. Specifically, maleimide groups are introduced to react with the thiol groups on the membrane of M2‐type macrophage‐derived exosomes, and these exosomes are further modified with positively charged oligolysine and polyethylene glycol (PEG). On one hand, PEG can prolong the exosomes’ blood circulation time, thereby enabling their accumulation at inflamed joints; on the other hand, M2‐type macrophage‐derived exosomes themselves carry miRNAs and cytokines, and possess a potent ability to chelate and clear cell‐free DNA.^[^ ^30^ ^]^

#### 3.3.2 Targeting Macrophages

**Figure 10:**
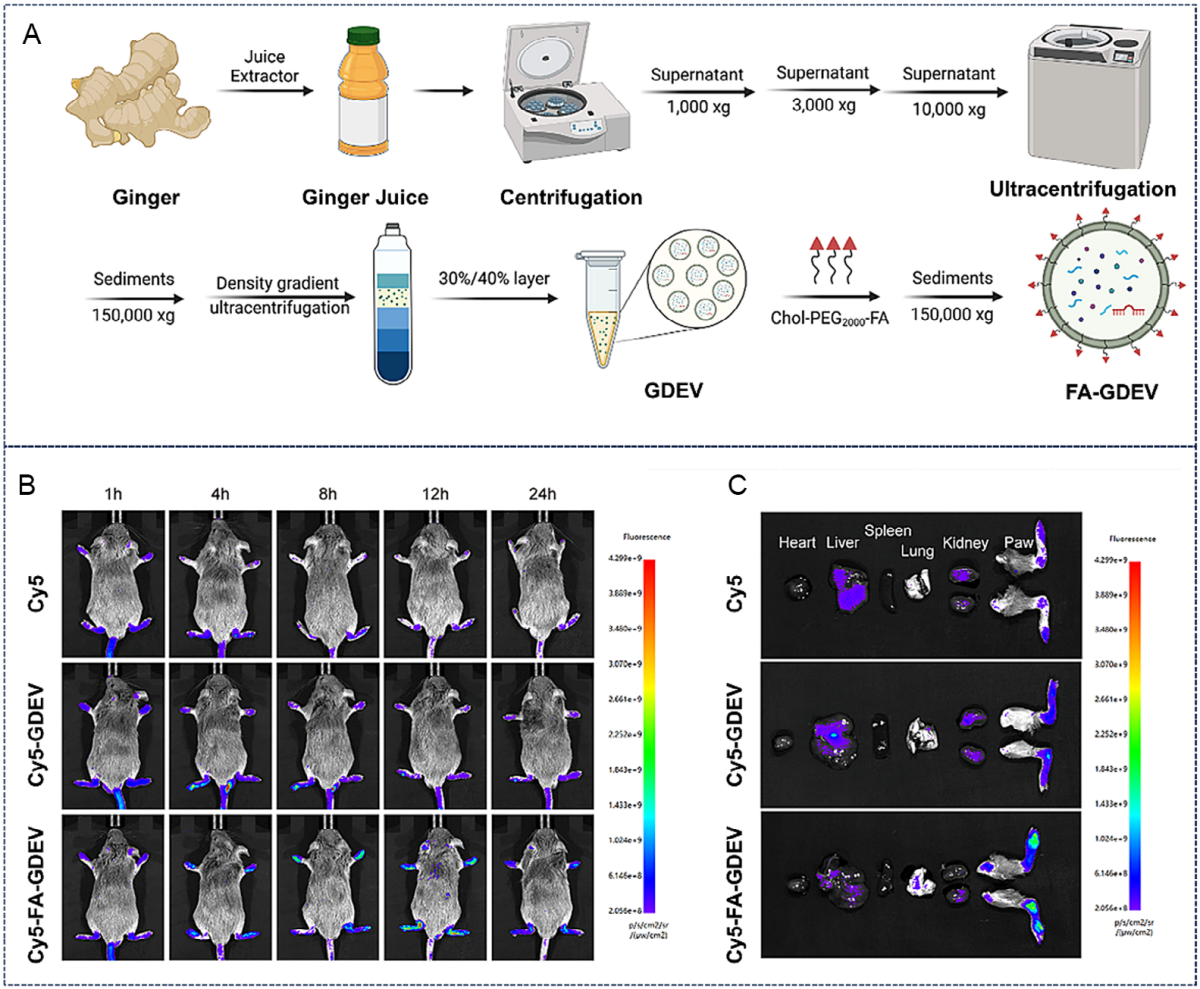
FA‐modified ginger‐derived exosomes target M1 macrophages and significantly alleviate RA symptoms. A) Schematic illustration of FA modification of ginger‐derived exosomes. B,C) FA‐modified ginger‐derived exosomes predominantly accumulate in joints following systemic administration. Reproduced with permission.^[^ ^87^ ^]^ Copyright 2025, John Wiley and Sons.

As mentioned earlier, FA can bind to folate receptors on the surface of macrophages. Taking advantage of this property, researchers have designed RA‐targeted exosomes directed at macrophages. Yan et al. successfully loaded dexamethasone sodium phosphate into folate‐modified exosomes via electroporation.^[^ ^42^ ^]^ The folate coating enhanced articular accumulation of dexamethasone‐loaded exosomes through active targeting of macrophage surface receptors, ultimately achieving significant reduction in joint inflammation levels and cartilage repair. Han et al. systematically investigated the therapeutic potential of ginger‐derived extracellular vesicles (GDEVs) for RA (**Figure** **10**A–C). By conjugating GDEVs with folic acid (FA), they developed FA‐GDEVs with intrinsic immunomodulatory properties.^[^ ^87^ ^]^ These FA‐GDEVs selectively targeted M1 macrophages in inflamed joints through folate receptors (FRs), promoting polarization toward the reparative M2 macrophage phenotype in vitro via modulation of the PI3K‐AKT pathway. The FA‐GDEVs not only ameliorated synovial and cartilage inflammation but also demonstrated the advantages of using natural plant‐derived exosomes for targeted RA therapy.

## 4 Conclusion and Perspective

With the intensification of global population ageing, cartilage‐related diseases such as OA, IVDD, and RA have emerged as major societal health threats, imposing substantial burdens on patients’ quality of life and healthcare resources.^[^ ^88^, ^89^, ^90^ ^]^ Conventional therapeutic approaches exhibit limited specificity and efficacy toward cartilage tissues, necessitating the urgent development of novel cartilage‐targeted strategies.^[^ ^88^, ^90^ ^]^ Exosomes, as natural nanovesicles, demonstrate unique advantages in drug delivery due to their excellent biocompatibility, stability, and robust capacity for transporting bioactive molecules, providing a promising strategy for therapy of cartilage‐related disorders.^[^ ^91^, ^92^, ^93^ ^]^

This review systematically summarizes exosome‐based cartilage‐targeted therapeutic approaches, with particular emphasis on construction strategies and disease applications. Regarding construction strategies, two primary methodologies exist: direct exosome modification and indirect parental cell engineering. Direct modification involves conjugating cartilage‐targeted ligands—including peptides (CAP, WYRGRL, etc.), cationic substances (avidin, cationic amino acids, chitosan), compounds (folic acid, chondroitin sulfate), nucleic acid aptamers, and antibodies—onto exosome surfaces. Indirect parental cell engineering encompasses genetic modification and click chemistry‐based approaches. Genetic engineering integrates genes encoding cartilage‐targeted proteins or peptides into the genome of exosome‐producing cells, thereby endowing exosomes with targeting capabilities. Click chemistry modification introduces unnatural azide groups onto cell surfaces via metabolic glycan engineering, followed by covalent conjugation of targeting molecules through click reactions to generate exosomes with specific homing properties. In disease applications, cartilage‐targeted exosomes have demonstrated remarkable therapeutic efficacy. For OA treatment, they effectively alleviate inflammation, promote cartilage repair and regeneration, and modulate gene expression by delivering anti‐inflammatory drugs, growth factors, and gene‐editing tools. In IVDD, targeted delivery to CEPs and nucleus pulposus cells improves cellular microenvironments and delays degenerative progression. For RA, macrophage‐targeted exosomes enhance articular accumulation of anti‐inflammatory agents, mitigating inflammation and facilitating cartilage repair.

Despite their immense potential, cartilage‐targeted exosomes face several challenges. Large‐scale production and purification techniques require optimization to enhance yield and quality for clinical translation.^[^ ^94^, ^95^ ^]^ Certain specialized delivery, such as gene therapies, remains cost‐prohibitive, limiting their application potential.^[^ ^96^ ^]^ Furthermore, long‐term safety profiles and potential side effects warrant thorough evaluation to ensure clinical reliability.^[^ ^97^ ^]^

Both direct modification of exosomes and indirect engineering of parental cells exert impacts on the exosomes themselves. Direct surface modification of exosomes leads to reduced yield due to multiple rounds of ultrafiltration. In contrast, indirect engineering of parental cells does not significantly decrease exosome yield, making it superior to direct surface modification in this regard.^[^ ^12^, ^14^ ^]^ Future research is likely to focus on improving the industrialization process of direct modification—optimizing ultrafiltration or chromatography procedures(such as tangential flow filtration) to reduce the loss of exosome yield.^[^ ^98^ ^]^ Furthermore, the therapeutic efficacy and safety of exosome modification remain concerns that require attention. For instance, if the insertion site of targeting peptides is located in the transmembrane domain of membrane proteins, it may cause abnormal membrane fluidity and impaired structural integrity of exosomes, thereby triggering cargo leakage and reducing their intrinsic biological activity.^[^ ^99^ ^]^ Although these modification methods have been proven safe in research, there is a lack of standardized protocols to ensure consistency in therapeutic efficacy. In the subsequent clinical application phase, unified standards should be established to set new requirements for modifiers, chemical coupling agents, payload dosages, and safety. For example, it is necessary to perform modifications on avidin to avoid immune responses, and appropriately reduce the number of peptides on the surface of individual exosomes to prevent off‐target effects.

The biosafety issues of genetically modified engineered exosomes remain a key concern in the field of exosomes. After viral or plasmid vectors carrying target genes are integrated into parent cells, residual DNA may be left behind. This residual DNA could integrate into the genome of parent cells, triggering abnormal biological processes in the cells.^[^ ^100^ ^]^ Exosomes produced by such “problematic cells” pose new risks when used in therapeutic applications. To avoid this risk, future research should improve the detection procedures for residual DNA and ensure that engineered exosomes are only produced when the residual vector DNA meets the required standards.^[^ ^101^ ^]^ For instance, the Chinese Pharmacopoeia (2020 Edition) stipulates that the residual DNA from host cells in biological products shall not exceed 100 pg per dose.

From the perspective of modification costs, antibody modification is costly. Antibody production relies on mammalian cell culture or hybridoma technology, which requires expensive bioreactors, serum‐free media, and strict sterile conditions.^[^ ^102^ ^]^ Its transportation and storage depend on cold‐chain logistics. Additionally, there are extra costs for endotoxin removal and glycosylation control.^[^ ^103^ ^]^ Thus, it is difficult for antibody modification to become mainstream for cartilage‐targeted modification. The cost of peptide modification depends specifically on sequence length and complexity (e.g., disulfide bonds). For instance, the peptide WYRGRL contains hydrophobic amino acids such as tryptophan (Trp) and tyrosine (Tyr), which makes its synthesis cost much higher than that of CAP.^[^ ^19^, ^20^, ^21^, ^22^, ^23^ ^]^ Yet, considering the difficulties in storage and synthesis, the cost of peptides is still lower than that of antibodies. Aptamer modification is relatively low‐cost. SELEX enables the in vitro synthesis of single‐stranded nucleic acids in a short period, and synthetic chemistry does not require the involvement of living cells.^[^ ^104^ ^]^ As nucleic acids, aptamers inherently possess stability and do not need special storage or transportation conditions.^[^ ^105^ ^]^ In the future, aptamer modification may become one of the most important modification methods.

Future research breakthroughs may overcome current technical limitations, improve safety, and reduce costs, ultimately facilitating the transition of cartilage‐targeted exosomes from laboratory research to clinical practice. Such advancements could provide more effective therapeutic options for patients with cartilage‐related diseases, improving quality of life while alleviating societal healthcare burdens.

## Conflict of Interest

The authors declare no conflict of interest.
